# Development of cyclic shedding teeth from semi-shedding teeth: the inner dental arcade of the stem osteichthyan *Lophosteus*

**DOI:** 10.1098/rsos.161084

**Published:** 2017-05-17

**Authors:** Donglei Chen, Henning Blom, Sophie Sanchez, Paul Tafforeau, Tiiu Märss, Per E. Ahlberg

**Affiliations:** 1 Department of Organismal Biology, Uppsala University, Norbyvägen 18A, 752 36, Uppsala, Sweden; 2 SciLifeLab, Uppsala University, Norbyvägen 18A, 752 36, Uppsala, Sweden; 3 European Synchrotron Radiation Facility, 6 rue Jules Horowitz, 38043 Grenoble Cedex, France; 4 Estonian Marine Institute, University of Tartu, Mäealuse Street 14, 12618 Tallinn, Estonia

**Keywords:** stem osteichthyans, tooth replacement, inner dental arcade, evolution of gnathostome dentition, three-dimensional palaeohistology, synchrotron microtomography

## Abstract

The numerous cushion-shaped tooth-bearing plates attributed to the stem group osteichthyan *Lophosteus superbus*, which are argued here to represent an early form of the osteichthyan inner dental arcade, display a previously unknown and presumably primitive mode of tooth shedding by basal hard tissue resorption. They carry regularly spaced, recumbent, gently recurved teeth arranged in transverse tooth files that diverge towards the lingual margin of the cushion. Three-dimensional reconstruction from propagation phase-contrast synchrotron microtomography (PPC-SRµCT) reveals remnants of the first-generation teeth embedded in the basal plate, a feature never previously observed in any taxon. These teeth were shed by semi-basal resorption with the periphery of their bases retained as dentine rings. The rings are highly overlapped, which evidences tooth shedding prior to adding the next first-generation tooth at the growing edge of the plate. The first generation of teeth is thus diachronous. Successor teeth at the same sites underwent cyclical replacing and shedding through basal resorption, producing stacks of buried resorption surfaces separated by bone of attachment. The number and spatial arrangement of resorption surfaces elucidates that basal resorption of replacement teeth had taken place at the older tooth sites before the addition of the youngest first-generation teeth at the lingual margin. Thus, the replacement tooth buds cannot have been generated by a single permanent dental lamina at the lingual edge of the tooth cushion, but must have arisen either from successional dental laminae associated with the individual predecessor teeth, or directly from the dental epithelium of these teeth. The virtual histological dissection of these Late Silurian microfossils broadens our understanding of the development of the gnathostome dental systems and the acquisition of the osteichthyan-type of tooth replacement.

## 1. Introduction

*Lophosteus superbus* Pander from the Late Silurian (Pridoli) of Ohesaare cliff, Saaremaa, Estonia, was described in 1856 and was thus among the first Silurian vertebrates to be studied [1]. It is represented by a rich but disarticulated material of scales, spines, dermal plates and tooth-bearing elements [2–4]. *Lophosteus* has been documented worldwide during recent decades. However, teeth and tooth-bearing bones are only known from the type locality material of *L. superbus*, while the other new species are mainly based on scales, spines and head plates [4]. Despite its fragmentary nature, *Lophosteus* has continued to attract the interest of researchers to the present day, partly because of the superb histological preservation of the Ohesaare material, and partly because of its distinctive character complement that suggests membership of the osteichthyan stem group.

For many decades, the earliest and most basal osteichthyans (bony fishes plus tetrapods) were interpreted as either actinopterygians (ray-finned fishes) or sarcopterygians (lobe-finned fishes), leaving the osteichthyan stem group vacant [5]. However, in 2007, Botella *et al*. [6] described marginal jawbones of the Silurian fishes *Andreolepis* and *Lophosteus,* and interpreted them as potential members of this stem group. In 2010, Friedman & Brazeau [5] established a synapomorphy scheme for the osteichthyan stem, crown and total group, and placed several Silurian–Devonian osteichthyans in the osteichthyan stem, but most of these are ‘scale taxa’ represented by very fragmentary material. Their scheme also proved controversial; Schultze [7] revised the scale characters of these taxa and argued that *Lophosteus*, interpreted by Friedman and Brazeau as a possible (but not definite) stem osteichthyan, is in fact the only known member of the osteichthyan stem group.

Besides scales and dermal plates, another common type of detached material that carries phylogenetic significance is dental elements. Osteichthyans have a unique mode of tooth replacement involving shedding through basal resorption of the dental tissues [8]. Gross identified *Lophosteus* as an osteichthyan rather than an acanthodian on the basis of detached teeth that showed traces of resorption and resembled crown osteichthyan teeth in the shape of their pulp cavities and arrangement of dentine tubules [2,3,9]. However, Friedman and Brazeau used almost no dental-development characters in their synapomorphy scheme, except the presence of enamel or acrodin [5], probably because of the dearth of data on the dental systems of stem osteichthyans.

The study of early vertebrate dental systems has recently been revolutionized by the application of propagation phase-contrast synchrotron microtomography (PPC-SRµCT), which allows the internal architecture of dental elements, including features such as buried resorption-overgrowth surfaces, to be visualized in three dimensions with micrometre-scale resolution without damaging the specimens [10,11]. Using this technique, a dentition field showing cyclical tooth replacement by basal resorption and shedding was discovered on a marginal jawbone of *Andreolepis* [12]. The shedding teeth of *Andreolepis* do not stand in longitudinal rows, but instead form a tooth field with an undulating labial boundary. They are most probably arranged in alternate transverse files, following the pattern of a more labial set of tooth-like non-shedding odontodes, which seem to have been used for food capture prior to the development of the shedding dentition. A well-organized linear longitudinal tooth row, with repeated basal resorption of the shedding teeth, is classically regarded as unique to osteichthyans. The dental development of *Andreolepis* suggests that the shedding cycle evolved before the emergence of the linear tooth row [12].

The Ohesaare material of *Lophosteus superbus*, which has been greatly expanded by a recent collecting programme led by H.B. includes marginal jawbones [6] and numerous small cushion-shaped tooth plates that Gross interpreted as ossicles from the hyoid arch or branchial arches in the oral cavity [3]. Similar cushion-shaped plates occur together with marginal jawbones in the material of *Andreolepis* as well [9,13]. The significance of these elements has recently been placed in a new light by the discovery of the so-called maxillate placoderm *Entelognathus* from the Silurian of Yunnan, China, which combine an overall placoderm-like morphology with marginal jawbones similar to those of osteichthyans [14]. Traditionally, it has been argued that the gnathal plates of conventional placoderms are homologous with the inner dental arcade (the coronoids, dermopalatines, ectopterygoids and vomers) of osteichthyans. However, *Entelognathus* and a second recently discovered form from the Silurian of Yunnan, *Qilinyu*, which both have maxilla, premaxilla and dentary, have no inner dental arcade at all [15]. This suggests that the gnathal plates are homologous with the outer dental arcade of osteichthyans, and that the inner arcade may be an osteichthyan novelty. The cushion-shaped plates of *Lophosteus* and *Andreolepis*, referred to below as ‘tooth cushions’, are certainly internal to the margins of the jaws and could thus, in principle, represent either the inner dental arcade, that is to say the coronoid-dermopalatine series, or still more internal elements such as branchial dental plates, or both. We return to this question in the Discussion, where we argue that they are more likely to belong to the inner dental arcade.

We present here the first investigation of the tooth cushions of *Lophosteus* by PPC-SRμCT at submicrometre resolution. As already illustrated in *Andreolepis* [12]. This groundbreaking technique reveals an extremely complicated three-dimensional histology, not recognized in the previous histological study by Gross [3], which allows the sequence of tooth addition and replacement to be inferred (electronic supplementary material, movie 1).

## 2. Material and methods

Fallen blocks of limestone totalling several hundred kilograms were collected from the type locality of *Lophosteus*, Ohesaare cliff, in Saaremaa Island, Estonia. Acetic acid dissolution and extraction of the microremains were, respectively, carried out at the Department of Earth Sciences of Lund University and the Department of Organismal Biology of Uppsala University, Sweden. All specimens are registered to the Geological Institute, Tallinn, Estonia under the designation GIT 760.

More than 130 tooth cushions were found among the microremains, displaying variation in size and morphology. The specimens can be confidently assigned to *Lophosteus* on the basis of a characteristic bone histology with very large osteocyte lacunae that is shared with scales, spines and bones of *Lophosteus* from the same locality [2,16]. The other gnathostomes in the fauna are acanthodians [17], which can be distinguished from *Lophosteus* without difficulty.

**Figure 1.**
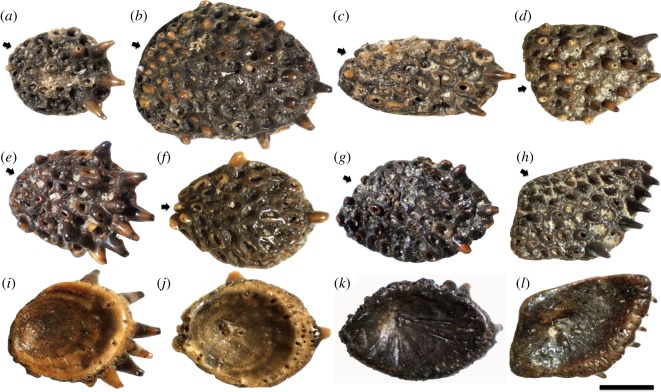
Morphological variation: (*a*) GIT 760-4, the smallest; (*b*) GIT 760-5, the biggest; and (*c*) GIT 760-6, the most elongated specimen. The tooth cushions are typically oval in shape (*e*,*f*: GIT 760-8, 760-9), sometimes with a straightened labial edge (*g*: GIT 760-10), and exceptionally triangular (*d*: GIT 760-7) or rhombic (*h*: GIT 760-11). Short arrow, origin of initial teeth and direction of median tooth files. (*i*–*l*) The basal view of (*e*–*h*), flipped vertically for orientation consistency. Lingual to the right, Scale bar, 1 mm.

**Figure 2.**
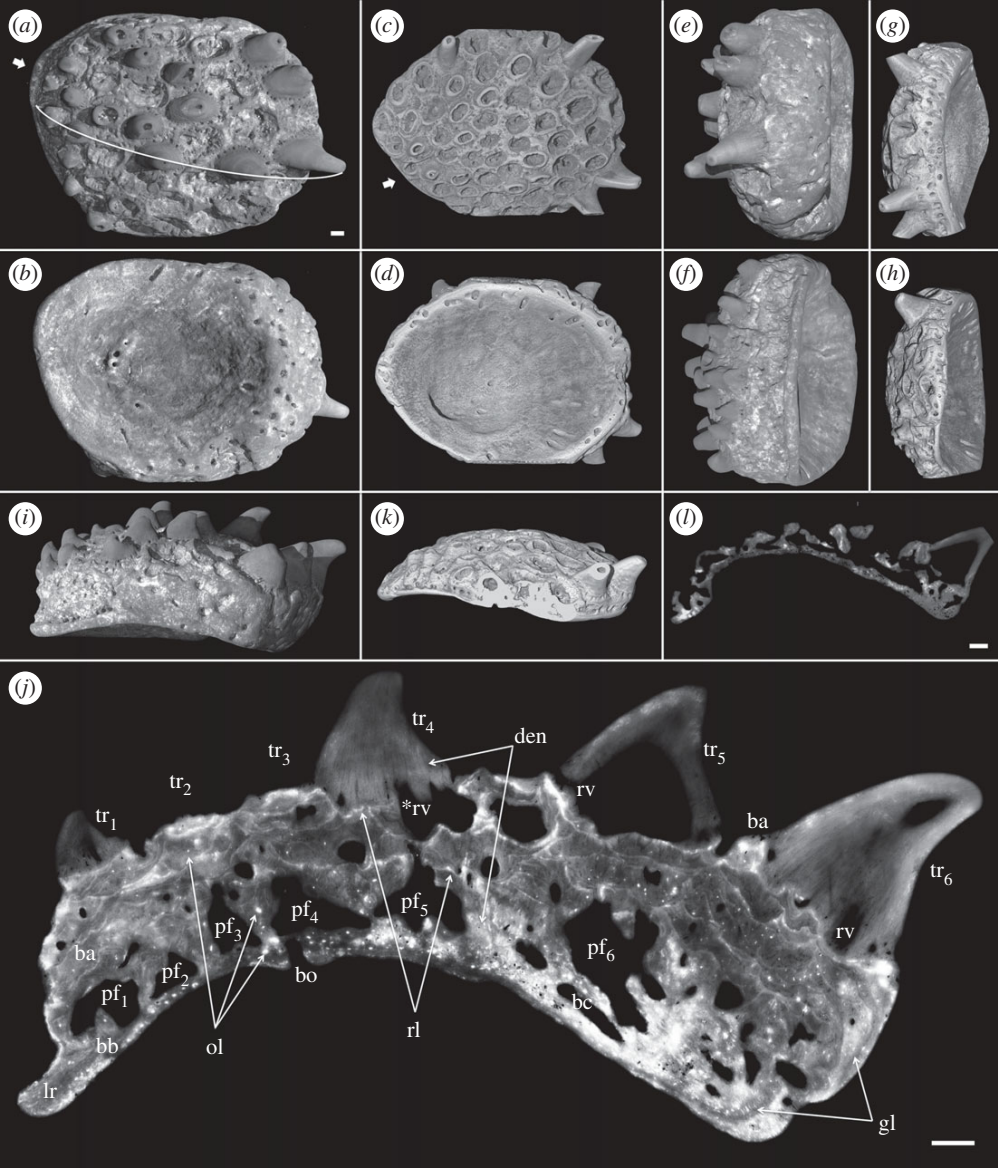
Surface renderings and virtual thin sections of two tooth cushions putatively from adult (GIT 760-1: *a*,*b*,*e*,*f*,*i*,*j*) and juvenile (GIT 760-2: *c*,*d*,*g*,*h*,*k*,*l*), based on PPC-SRμCT data. (*a*,*c*) External, (*b*,*d*) internal, (*e*,*g*) lingual, (*f*,*h*) labial, (*i*,*k*) lateral views and (*j*,*l*) transverse sections through a tooth file. The section plane of (*j*) is shown by a curving white line in (*a*). (*a*–*d*,*i*,*j*) lingual to the right, (*b*,*d*) are flipped vertically for orientation consistency. Short arrow, origin of initial teeth and direction of median tooth file. Scale bars, 0.1 mm, (*a*–*i*,*k*) to the same scale. Abbreviations are given in §3.1.

**Figure 3.**
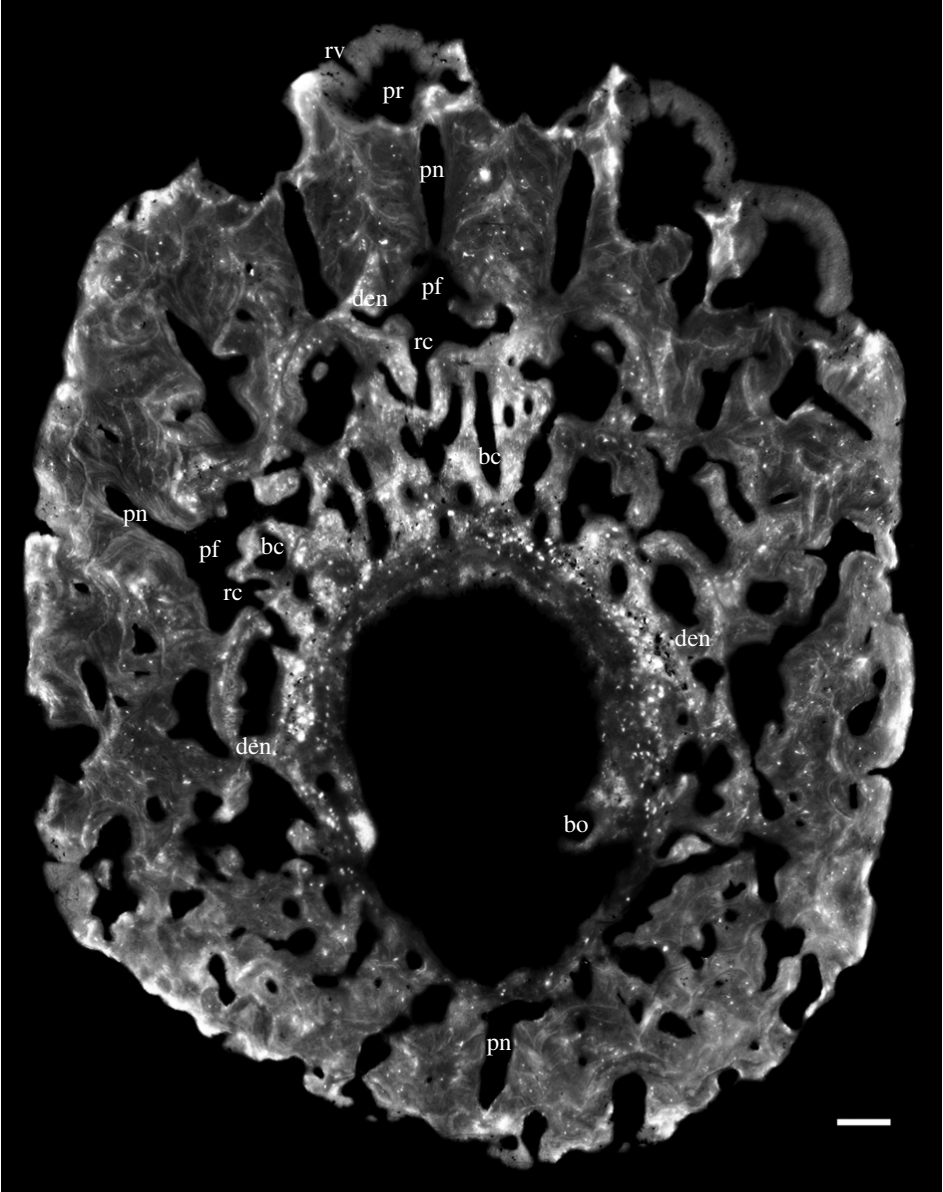
Horizontal virtual thin section of the adult specimen (GIT 760-1). Lingual at top. Scale bars, 0.1 mm.

**Figure 4.**
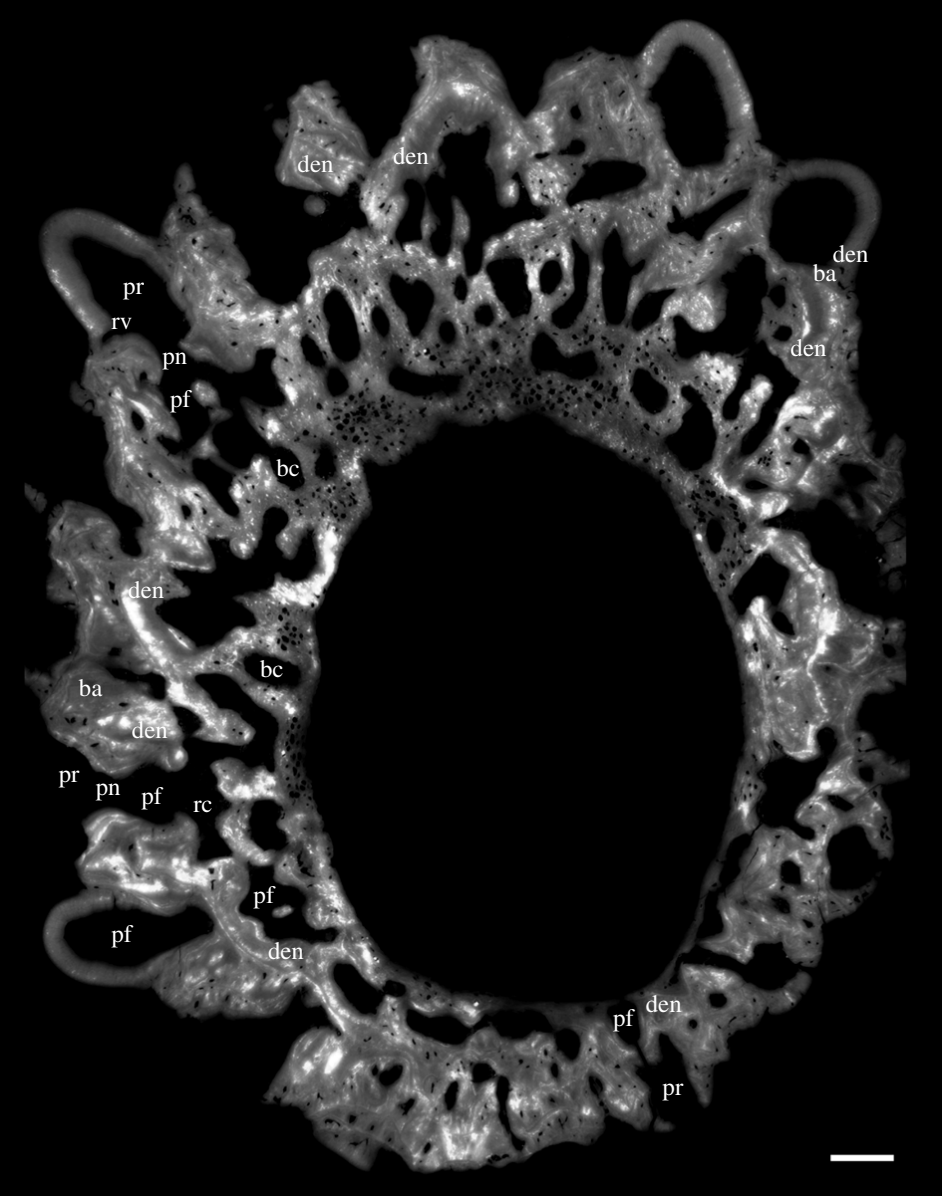
Horizontal virtual thin section of the juvenile specimen (GIT 760-2). Lingual at top. Scale bars, 0.1 mm.

**Figure 5.**
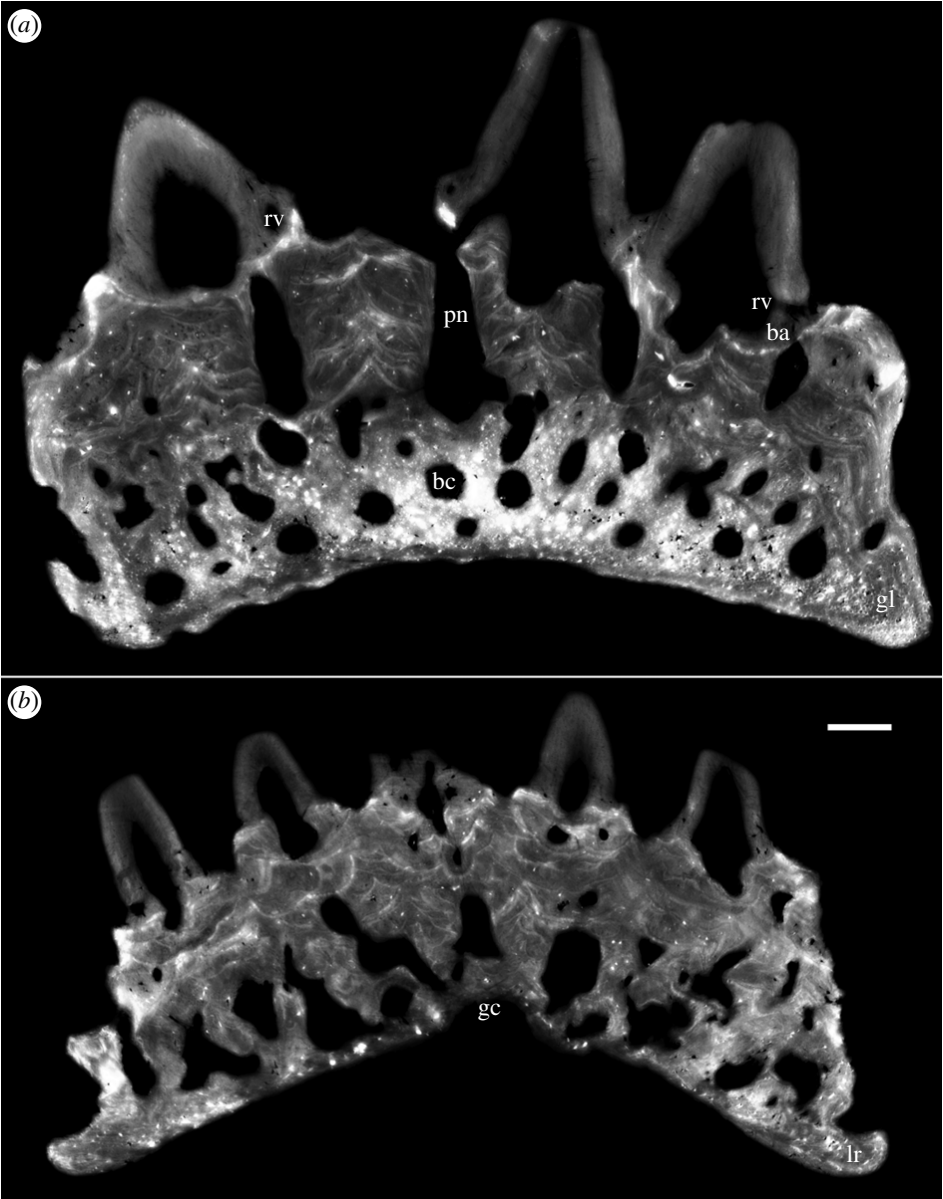
Longitudinal virtual thin sections of the adult specimen (GIT 760-1). (*a*) Section close to the lingual margin and (*b*) section close to the growth centre of the tooth cushion. Scale bars, 0.1 mm, (*a*,*b*) to the same scale.

**Figure 6.**
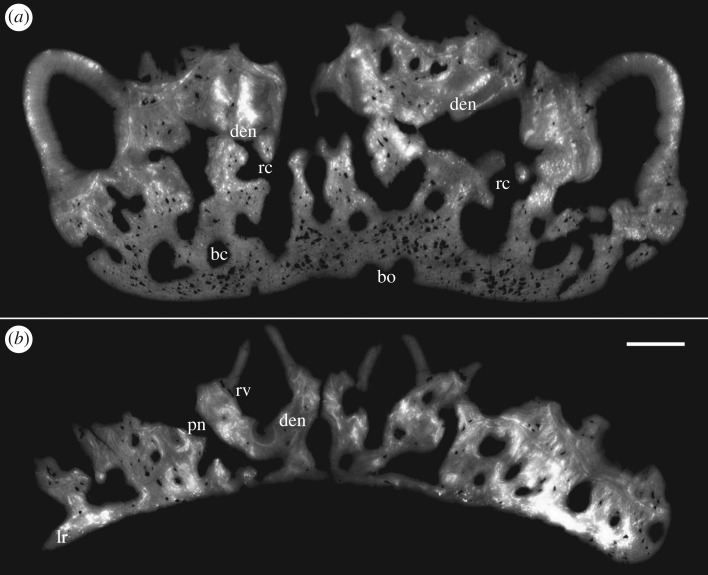
Longitudinal virtual thin sections of the juvenile specimen (GIT 760-2). (*a*) section close to the lingual margin and (*b*) section close to the growth centre of the tooth cushion. Scale bars, 0.1 mm, (*a*,*b*) to the same scale.

**Figure 7.**
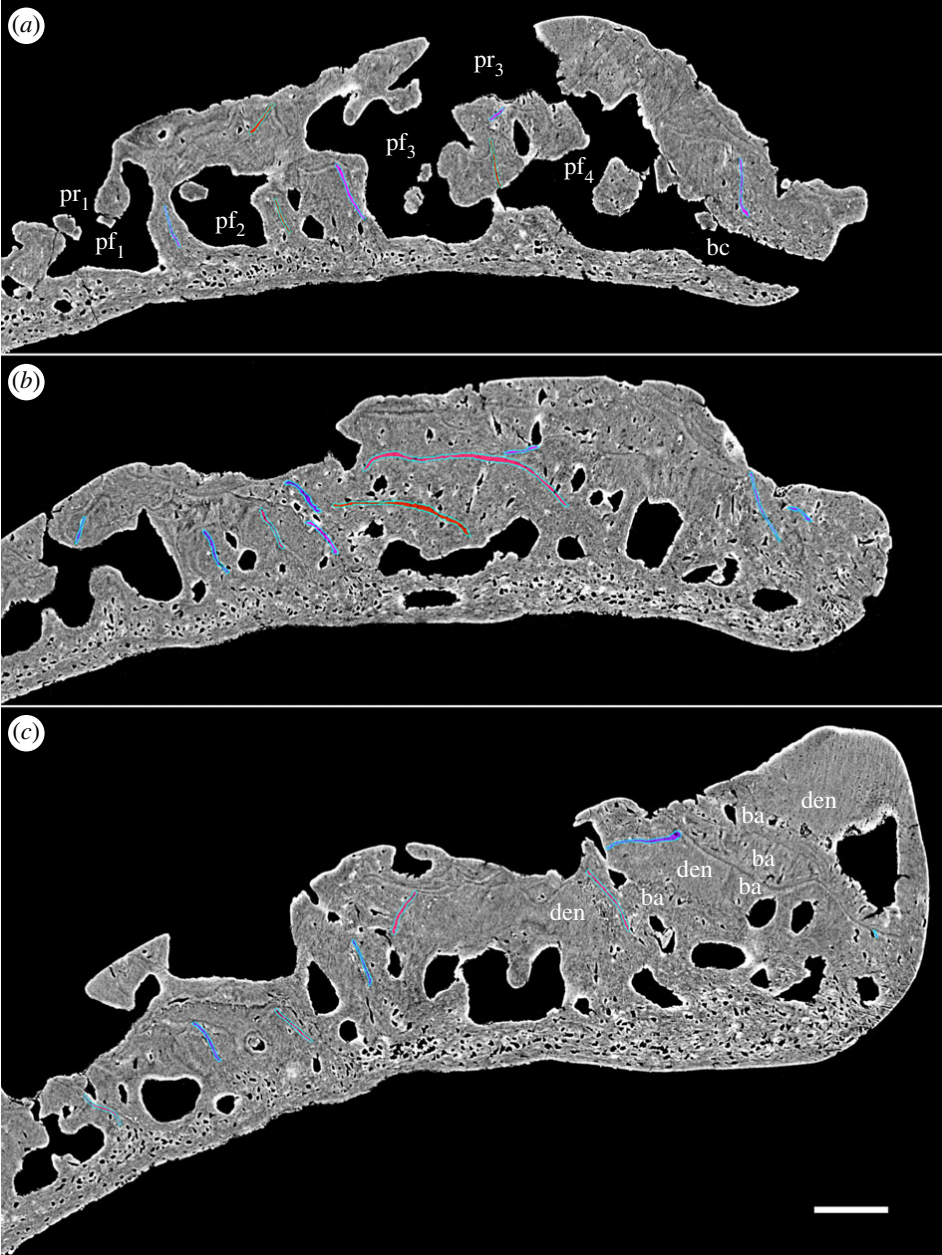
First-generation tooth files of GIT 760-3 in (*a*,*c*) transverse virtual thin sections of two files, respectively, and (*b*) through the overlap zone at the border between the two files. Lingual to the right. Scale bar, 0.1 mm, (*a*–*c*) to the same scale. Colour codes are given in §3.2.

**Figure 8.**
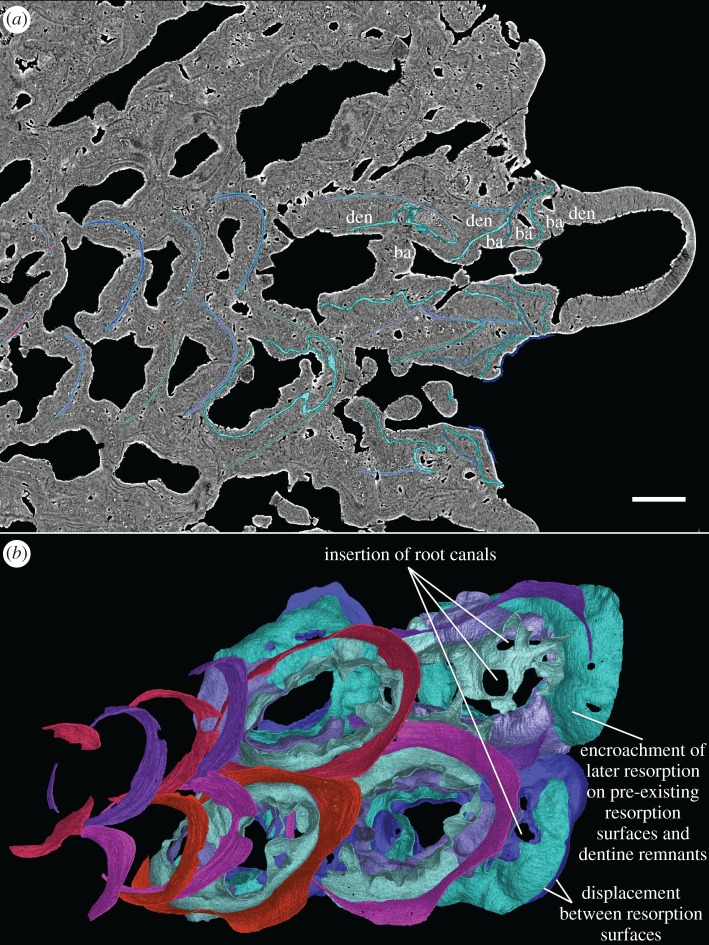
Reconstruction of embedded structures from two to three dimensions, exemplified by two first-generation tooth files of GIT 760-3. (*a*) Horizontal virtual thin section. (*b*) Visualization of the dentine rings of the first-generation teeth and stacks of resorption surfaces in three dimensions, as the internal view of figure 9*f*. Lingual to the right. Scale bars, 0.1 mm, (*a*,*b*) to the same scale.

Ohesaare cliff represents an open shoal environment with high energy [18], and many of the *Lophosteus* tooth cushions show evidence of transportation and abrasion. Most tooth cusps were broken, leaving a cluster of rings of the dentinal bases, and thus the cushions acquired a honeycomb appearance (figures 1*a*–*h* and 2*a*,*c*). But this has no influence on the excellent preservation of histology, which yields a wealth of details and allows the growth history to be reconstructed with confidence. Dentine and bone can easily be distinguished and there is no visible recrystallization of either tissue (figures 2*j*–8*a* and 11*a*). Vascular canals, osteocyte lacunae, dentine tubule and fibre traces are all clearly visible and show no evidence of distortion or enlargement by post-mortem erosion.

### 2.1. Focus-stack imaging

Selected specimens were photographed by dissection microscope each at 20–30 focus points. These separate frames were combined by the Auto-Blend Layers command of Photoshop to composite an image in which the whole specimen is in focus (figure 1).

### 2.2. PPC-SRµCT

Three specimens were imaged at beamline ID19 of the European Synchrotron Radiation Facility (ESRF) in Grenoble, France, using propagation phase-contrast synchrotron radiation microtomography (PPC-SRµCT) adapted to fossil bone histology [10,19] (table 1).

**Table 1.** List of the technical parameters of the synchrotron scans.

sample number	GIT 760-1	GIT 760-2	GIT 760-3
voxel size (µm)	0.696	0.696	0.678
optic	ID19 revolver objective10× 0.3NA + eyepiece 2×	ID19 revolver objective10× 0.3NA + eyepiece 2×	ID19 revolver objective10× 0.3NA + eyepiece 2×
date	07 July 2014	05 July 2014	29 August 2009
average energy (keV)	19	19	30
filters (mm)	Al 0.7	Al 0.7	Al 2
propagation distance (mm)	15	15	30
monochromator	none	none	single bounce W/B4C multilayer 2.5 nm period
sensor	FReLoN 2K14	FReLoN 2K14	FReLoN 2K14
scintillator	GGG : Eu 10 µm	GGG : Eu 10 µm	GGG : Eu 10 µm
insertion device	U17.6 ID19	U17.6 ID19	U32 ID19
ID gap (mm)	20	20	12.38
projection number	4998	2499	2000
references every N proj	4998	2499	200
scan geometry	360° half-acquisition 400 pixels	360°	180°
exposure time (s)	0.3	0.3	0.3
time per scan (min)	38.27	19.93	18.86
number of scans	1	1	1
reconstruction	paganin	paganin	edge detection

**Figure 9.**
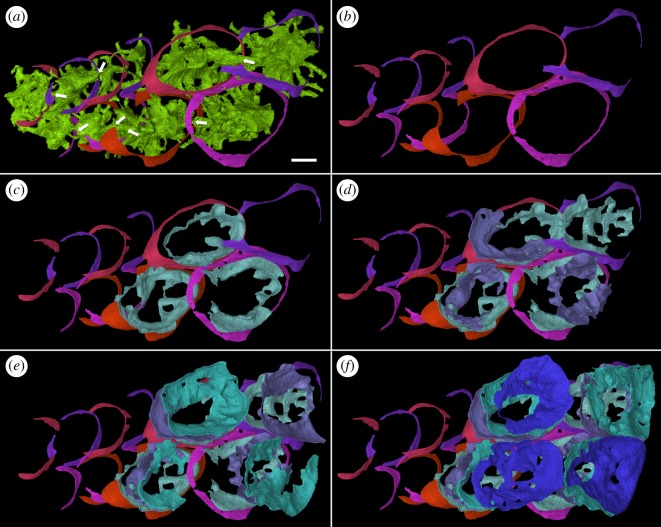
Virtual dissections in external view visualizing two first-generation tooth files and overlying stacks of resorption surfaces in tooth cushion GIT 760-3. (*b*) Rings of dentine remnant that overlap each other, shown with (*a*) their pulp cavities, and (*c*–*f*) resorption surfaces of four rounds of shedding at the four largest tooth sites. Note that each tooth position moves lingually during ontogeny so that the stacks of resorption surfaces become oblique. Arrow, the connections between the first-generation pulp cavities above the dentine rings. Lingual to the right. Scale bars, 0.1 mm, (*a*–*f*) are to the same scale.

**Figure 10.**
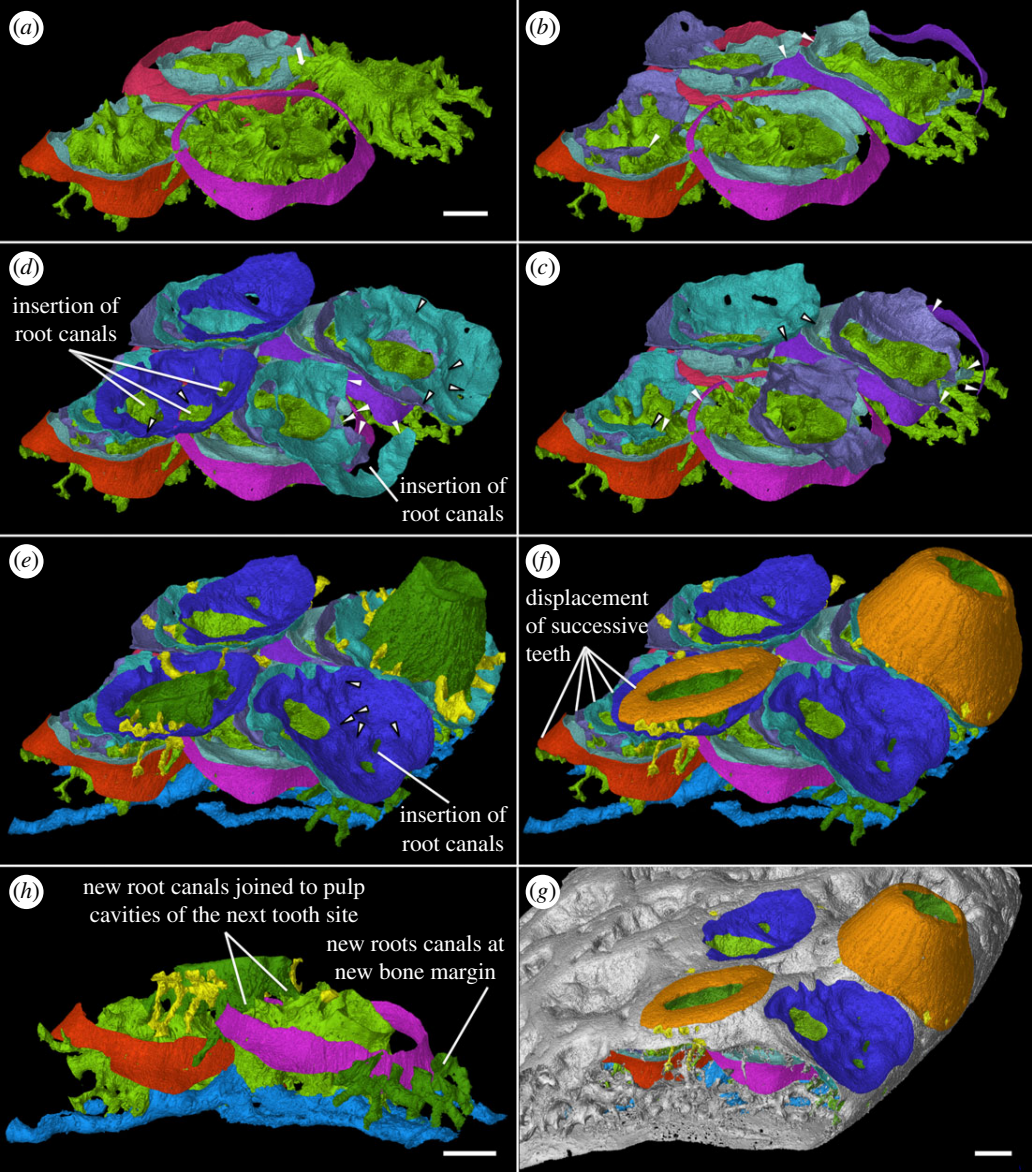
Oblique external-lateral close-ups of tooth addition and cyclic resorption of the four youngest tooth sites of two alternate files in tooth cushion GIT 760-3. (*a*) The first-generation teeth had undergone semi-basal resorption before the next addition, and the pulp cavities of new teeth join the old ones by crossing the dentine rings. (*b*–*d*) Successive replacement teeth are shed by basal resorption, leaving resorption surfaces stacked on top of the first-generation tooth remnants. (*e*) The rest of the vascular system, including the large canals of the basal bone, the radial vessels of the pulp cavities and the pulp cavities of the function teeth are shown. (*f*) The dentine crowns of the functional teeth are shown. (*g*) The surrounding bone and vascular surface are shown. (*h*) The lateral view of the canal system of two neighbouring tooth sites in one tooth file. Arrow, the connections between the first-generation pulp cavities above the dentine rings. Arrow head, encroached structures by later resorption events. Arrow head with black outline, resorption events that affect the structures indicated by white arrow heads in the previous figures. Lingual to the right. Scale bars, 0.1 mm, (*a*–*f*) are to the same scale.

**Figure 11.**
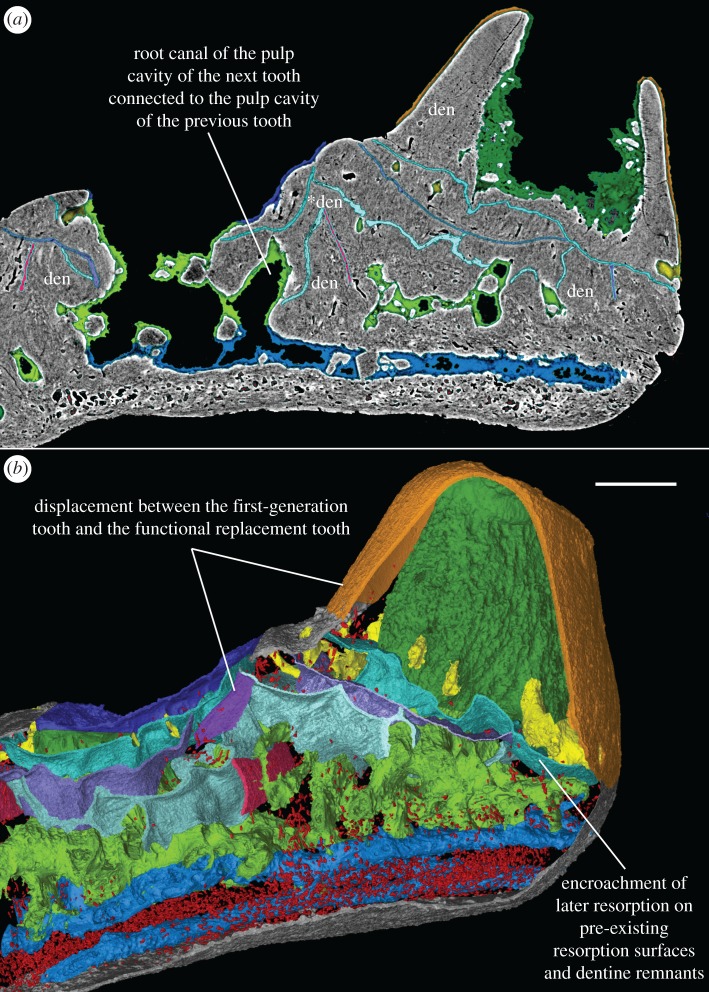
Transverse virtual thin section and virtual slab through two tooth sites in a file, showing the distribution of osteocyte lacunae. Lingual to the right. Scale bars, 0.1 mm, (*a*,*b*) are to the same scale.

The scan that the three-dimensional virtual dissection (figures 7–11) was based on was done on specimen GIT 760-3 with a voxel size of 0.678 µm. It was obtained with an objective 10×, NA0.3 coupled with a 2× eyepiece. The optics, associated with a gadolinium gallium garnet crystal of 10 µm thickness (GGG10) scintillator, is coupled to a FreLoN 2K14 detector (fast readout low noise camera [20]). The sample was set up at a distance of 30 mm from the optics. The experiment was performed with a monochromatic beam obtained thanks to the use of a single crystal 2.5 nm period W/B4C multilayer monochromator fixed to the energy of 30 keV. The gap of the undulator of the insertion device (U32U) was set to 12.38 mm. During the scan, 2000 projections were done over 180° with a time of exposure of 0.3 s. The scanning data were reconstructed using a classical filtered back-projection algorithm (PyHST software, ESRF) adapted to local tomography [21]. This was done in edge-detection mode, based on an assumption of chemical homogeneity. Artefacts (e.g. rings and movements) were corrected based on programmed filters.

Data for ontogenetic comparisons (figures 2–6) are from scans of two specimens, GIT 760-1 and GIT 760-2, with a voxel size of 0.696 µm (also obtained with an objective 10×, NA 0.3, coupled with a 2× eyepiece, a GGG10 scintillator, a FreLoN 2k14 detector and 0.3 s exposure time). The sample was set up 15 mm from the optics. The gap of the undulator U17.6 was set to 20 mm and provided a pink beam (direct beam with a single main narrow harmonic) at an energy of 19 keV. A total of 2499 projections were taken over 360° for the smaller specimen, or 4998 projections over 360° by half-acquisition 400 pixels for the larger one. Reconstruction was done with a modified version [10] of a single-distance phase retrieval approach [22].

The virtual thin sections of the sample in the form of stacks of images were segmented into three-dimensional sub-volumes through the software VG Studio 2.2. Embedded subtle structures, such as the surfaces of resorption and dentine, were traced manually.

## 3. A note on morphological terminology

The tooth cushion dentition of *Lophosteus* has a complex growth mode that includes both the addition of new teeth to the ends of existing tooth files, thus creating new tooth sites, and the repeated shedding and replacement of the teeth at existing sites. In order to express this organization unambiguously in writing, the following terminology is used throughout: tooth file—a linear arrangement of teeth, oriented labio-lingually with new teeth added at the lingual margin; first-generation tooth—the first tooth to grow at any tooth site; pioneer teeth—the earliest first-generation teeth; previous, next—terms denoting order of deposition of first-generation teeth within a file; tooth site—a location initially established by a first-generation tooth, later maintained by a replacement sequence of successor teeth; predecessor, successor—terms denoting time sequence of teeth within a replacement sequence at a single tooth site.

### 3.1. Abbreviations

ba, bone of attachment;

bb, bony base;

bc, radial canal of bony base;

bo, basal canal opening;

den, dentine (*den, the original surface of the dentine tissue in this area has been eroded by the resorption surfaces);

gc, growth center of bony base;

gl, growth line;

lr, labial rim;

ol, osteocyte lacunae;

pf, pulp cavity of the first-generation tooth;

pn, pulpal neck linking the pulp cavities of the first-generation and the functional replacement teeth;

pr, pulp cavity of the replacement tooth;

rc, root canal of the first-generation tooth;

rl, resorption line;

rv, ascending vessel radiating from pulp cavity (*rv, radial vessel connecting to the pulp cavity of neighbouring teeth);

tr, tooth of replacement (the numbers of pf and tr indicate the corresponding tooth sites between first-generation and replacement teeth).

### 3.2. Colour codes


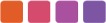
 first-generation teeth


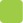
 first-generation pulp cavities


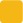
 replacement (functional) teeth


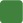
 replacement teeth pulp cavities


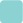
 semi-basal resorption surfaces


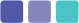
 basal resorption surfaces


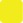
 radial vessels of pulp cavities


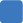
 radial canals of bony base


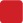
 osteocyte lacunae

## 4. Results

### 4.1. Morphological variations

The apex of the tooth cushion, surrounded by appositional growth increments (figures 1*i*–*l* and 2*b*,*d*), is believed to be the growth centre of the bony plate. The apex is not located at the centre of the tooth cushion, which grows faster in the (assumed) lingual direction. The labial edge of the tooth cushion is especially thin, free of vascular openings and tooth covering (figures 1, 2*a*,*c*,*f*,*h*,*i*–*l* and 5*b*, 6*b*,lr). It seems the tooth cushion is in contact with another bone (presumably a marginal jawbone) labially by a rim of basal bone, and the growth of bone in this direction is limited. The majority of the specimens are ovoid in shape, with the labial rim at the narrow end and the thicker vascularized edges at the broad end (figure 1*a,e*,*f*,*i*,*j*), but there are exceptions that are oriented the other way around (figure 1*b*,*d*). Sometimes the narrow end is pointed, or the labial rim is straightened (figure 1*g*,*k*); in some specimens, all the edges are straightened, making the tooth cushion approximately rhomboidal (figure 1*h*,*l*). Rarely, the tooth cushions may be further elongated into a rectangular shape (figure 1*c*). Most cushions are obviously (though not grossly) asymmetrical, but one specimen (figure 1*f*,*j*) is bilaterally symmetrical and could represent a symphysial plate.

Interestingly, the pioneer teeth are not situated above the growth centre of the bony plate (figures 1*e*–*l* and 2*a*–*d*). The oldest tooth sites are aligned along the labial rim (figures 1 and 2*a*,*c*, short arrow). If the labial rim is at the narrow end of the tooth cushion, the tooth files radiate lingually from there towards the thicker edges symmetrically (figure 1*f*); but the labial rim is more commonly at one side of the narrow end, and the tooth rows are arranged in an asymmetric pattern such that the direction of the median tooth file is not parallel to the long axis of the tooth cushion (figure 1*a*–*e*,*g*,*h*). Teeth are closely spaced and recurved towards the direction of addition (mostly lingually). New tooth files may originate between the diverging original tooth files, about halfway towards the broad end of the tooth cushion. New teeth become larger and larger as well. The number of tooth sites varies greatly among specimens, but not necessarily depending on size. The largest specimens can carry about 14 rows and 120 functional teeth (figure 1*b*), but both the smallest (figures 1*a* and 2*c*) and medium-sized ones (figures 1*c*–*h* and 2*a*) can carry a wide range, between 40 and 60 teeth.

### 4.2. Tissue organization

The general organization of the tooth cushion is that of a domed basal plate of bone, with a convex external surface and a concave internal surface, carrying on its external surface a tissue layer containing a complex array of multi-layered resorption features, sandwiched between two sets of teeth. The upper set comprises the functional teeth protruding from the external surface; the lower set consists of the retained bases of the first-generation teeth, lying directly on the bony base. Both sets contain the same number of tooth sites (figure 2*j*, pf_1–6_, tr_1–6_). The multi-layered resorption features separating the tooth sets are the product of repeated tooth shedding and replacement at these sites.

**Figure 12.**
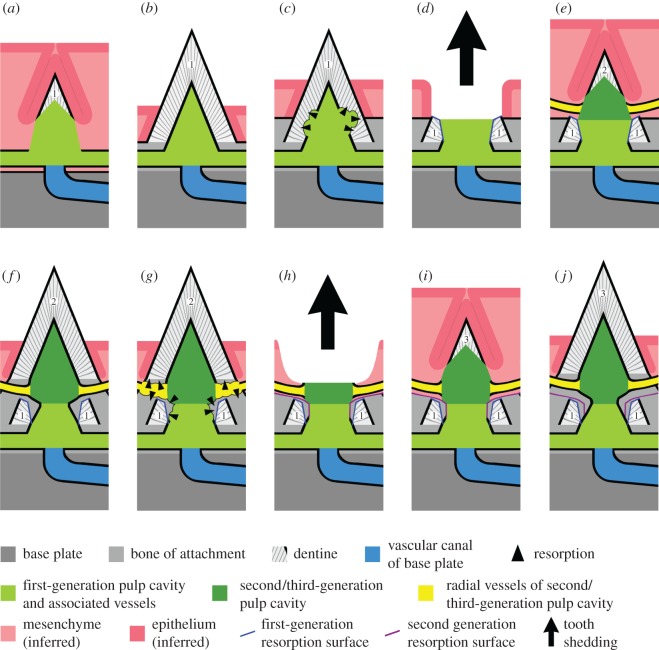
Schematic model of tooth replacement process, starting with the growth of a first-generation tooth (*a*,*b*) and passing through the shedding of this tooth (*c*,*d*), the growth of a replacement tooth (*e*,*f*), the shedding of this replacement tooth (*g*,*h*) and the growth of a second replacement tooth (*i*,*j*). Numbers identify the first, second and third tooth. The model aims to explain how the tooth replacement process created the spatial relationships of the resulting resorption surfaces and other structures, but in two important respects it is simplified relative to the actual tooth cushions. Firstly, it does not include a component of lateral displacement. Secondly, it shows the replacement tooth developing after its predecessor has been shed, whereas growth of the replacement tooth in reality probably started alongside the predecessor tooth before shedding.

The bony base contains numerous osteocyte lacunae (figures 2*j*, ol, 3–7 and 11, in red). Osteocyte lacunae are also found in lower numbers in the bone of attachment, a bone-like supportive tissue that cements the teeth into place. The resorption process that initiated tooth shedding did not remove all the bone of attachment, with the result that this tissue accumulated and thickened the tooth cushion with each tooth replacement cycle (figure 12). The gaps between the functional teeth (figures 2*j*, ba, 3–6 and 11), as well as the tooth-free margin (figure 2*f*,*i*,*j*, ba) are actually filled by stacks of the bone of attachment that surrounded preceding generations of shed teeth.

The teeth consist of orthodentine and lack enamel. The bone of attachment and dentine grade into each other in a transition zone without any sharp boundaries (figures 4, 7*c* and 8*a*, ba, den), consistent with their probable derivation from the same odontogenic population of mesenchyme cells within the dental papilla [23]. The transition zone, the basal dentine rich in cell lacunae, was described by Gross [2] as somewhat folded with fewer tubules and pierced by radial vessels.

After tooth addition and cyclical replacement, all the embedded old tissues, dentine and bone of attachment of the preceding generations of teeth, are integrated into the basal plate. The basal plate as a whole keeps growing wider and deeper, which can be traced by the growth arrest lines. The distinction between adult and juvenile is not the size, shape or number of functional teeth, but more likely the thickness of the tooth cushion and number of resorption surfaces in each stack (figures 2–6). New bony tissue is added to the cushion margin and the internal surface, but not the external surface after the start of tooth replacement. The bone-like tissue accumulated onto the external surface is bone of attachment, because its addition always follows the course of tooth replacement, creating an uneven external surface and the basal resorption surfaces all reach the external surface of the bone, if not encroached by others.

### 4.3. Three-dimensional architecture of vasculature

The imprint of large vascular canals can be found on the internal surface radiating from the apex of the tooth cushion to the bone margin (figures 1*i*–*l* and 2*b*,*d*), where they are truncated obliquely. There are at least two more layers of these radial basal canals running parallel to the curvature of the bone, inside the bony base and between the bony base and the tooth division (figure 11, sky blue). Similar to the longitudinal vascular mesh through the osteocyte-rich layer of the basal bone of *Andreolepis* [12], they split or unite uncommonly, but never cross perpendicularly. Their openings on the internal surface are concentrated at the bone margin and at the growth centre of the bone or somewhat lingual to it (figures 1*j*–*l* and 2*b*,*d*), with a scatter between these areas (figure 1*i*). We interpret them as housing the arteries and veins that supply the complex vascular system of the dental layer as well as the overlying soft tissue. A single pulp cavity (a tooth site) at the cushion margin can connect up to seven basal canals of the bony base by its root canals, and all the oval-shaped pulp cavities are elongated in the direction of the radial basal canals (figure 10*e*,*f*).

The pulp cavities of functioning replacement teeth (figures 9–11, dark green) have a rounded bottom sitting on top of the stack of resorption surfaces (figures 3–6), whereas those of the first-generation teeth are based on a complex network of root canals (figures 9*a*, 10 and 11, bright green) that radiates irregularly from the centre of the pulp cavity and joins different tooth sites together. These canals represent the vascular network that initially formed in mesenchymal tissue above and outside the edge of the bony base plate, during the earliest stage of tooth development and provided the blood supply for the tooth buds. The deposition of bone of attachment around and between these vessels during the later stages of tooth development caused them to become embedded in the tooth cushion and thus preserved (figure 12*a,b*). Occasional vertical canals rising from the root canals show that they remained connected to the vasculature of the overlying soft tissue.

Replacement teeth have a dual vascular supply with deep and superficial components. The deep component normally came up through the (now open-topped) pulp cavity of the predecessor tooth (figures 3, 5*a*, 7*a* and 11*a*). However, the youngest generations of replacement teeth are formed in a substantially more lingual position than their predecessors (figures 9–11), which is more obvious at the younger tooth sites of each radial file. Some replacement teeth have shifted to the level between two dentine rings, which mark the original position of the tooth sites (figure 10*d*–*f*), and show two separate sets of deep canals connecting to the first-generation pulp cavities of the two tooth sites (figures 7*a* and 10*h*). The replacement teeth at the youngest tooth sites may have one set of deep canals coming up through the marginal bone of the base plate (figure 10*e,f*,*h*), apart from the one arising out of the predecessor pulp cavity. In this instance, it appears that the site of the replacement tooth bud straddled the margin of the underlying base plate, such that it was able to attract vessels both from the predecessor pulp cavity and from the soft tissue bordering the base plate.

In addition to these deep components, each replacement tooth has a ring of more superficial vessels, represented by radial canals emerging from a higher level of the pulp cavity, which in life connected to the vasculature of the thin soft tissue overlying the tooth cushion (figures 2*j*, 3–6, rv; 10*e*–*h*, 11 and 12*e–j*, yellow). The openings of these canals lie within the bone of attachment and form a ring round each standing tooth, which also characterizes the shedding teeth on the tooth cushion of *Andreolepis*, and the marginal jawbones of *Lophosteus* and *Andreolepis* [12, 24]. The superficial canals closely parallel the surface of the immediately underlying resorption cup, and very few of them are entrapped in the remnant bone of attachment between resorption cups in a stack. This implies that the osteoclast activity that loosened the predecessor tooth from the cushion proceeded from these vessels. The tooth shedding process would thus always end with the superficial vessels lying free in soft tissue above the resorption cup, and irrespective of whether those actual vessels survived to be re-embedded by the bone of attachment of the successor tooth, or whether they were broken down and replaced by new vessels at a slightly higher level, they would not be captured between resorption surfaces (figure 12*e–j*). The few examples that were captured in this way presumably represent vessels that failed to initiate osteoclast activity. The openings are often shared by the radial vessels of neighbouring teeth. If the openings are covered by a new tooth of its own or adjacent tooth site, the radial canals will join to the bottom of the new pulp cavity (figure 2*j*, *rv). However, the few radial vessels belonging to the older generations of pulp cavities can still communicate with the soft tissue in places where there is no overgrowing of new teeth. The radial canals can also give off straight branches that connect down to the root canal network directly.

Gross [3] examined the shed teeth with resorption pits and described that the pulp cavities are undivided and unfolded, wide open at the base and narrowing upwards to a narrow channel, but he missed the resorption surfaces underneath the replacement teeth and the first-generation teeth buried in the basal plate. Because the first-generation teeth were shed semi-basally, that is to say by resorption proceeding from within the pulp cavity a little way above the base of the tooth (figure 12*c,d*), the bases of their pulp cavities together with the dentine became embedded by the bone of attachment of the following generations of teeth. The pulp cavities of the next generations lay on the pre-existing ones, connecting to the vascular networks of the bony base through the old chambers. But as all the younger generations of teeth were shed basally, their pulp cavities were not preserved. After the replacement of several generations of teeth and the stacking of bone of attachment, a neck is formed linking the pulp cavities of the first generation and those of the functional teeth (figures 3–6, pn), which is more obvious in the older tooth cushions that have undergone a greater number of tooth replacement cycles. Along with the growth of the basal plate, the teeth become bigger and tend to move towards the bone margin. Consequently, the column of the oldest and youngest pulp cavities and the neck is inclined towards the margin (figures 2*j*, 10 and 11).

### 4.4. Tooth addition, shedding and replacement

The overlapping relationship between the first-generation teeth shows that they are added sequentially towards the growing margin of the tooth cushion in an alternate pattern (figures 7–11). Although the origin of radial files (figures 1 and 2*a*,*c*, short arrow) does not coincide with the growth centre (the apex) of the bone, tooth addition correlates with the growth of the basal plate. Teeth are arranged so tightly that, in a radial row, a younger larger first-generation tooth overlaps the preceding tooth site for at least a quarter of the tooth length, and even overlaps on two preceding teeth. As a result, the preceding tooth has to be shed to make way for the next tooth, which allows the root canals of the new tooth to join those of the preceding tooth from above (figures 9*a*, 10*a* and 11). However, the first-generation teeth are shed semi-basally, that is to say, only the crown is lost (figure 12*a*–*d*). It leaves the periphery of the tooth base that, before shedding, has probably been incorporated into the basal plate, embedded in the bony tissue to some extent, as well as overlapped by a newly added tooth of the adjacent row. The marginal first-generation teeth, the final tooth sites of the tooth files, also undergo semi-basal resorption.

After the addition of the first-generation teeth is finished, the tooth cushion continues growing wider; however, the number of tooth sites does not increase. *In situ* tooth replacement is thus required to produce new sharp teeth on the surface of the tooth cushion. This has apparently already happened to the oldest tooth sites before the addition of the last first-generation teeth to the bone margin, because the larger number of resorption surfaces at the labial tooth sites documents that they have been replaced more times (figures 2*j*–6). Among the four tooth sites modelled in exhaustive detail, the functional tooth of the youngest tooth site is one generation younger than that of the oldest tooth site (figures 9*c*–*f* and 10*e*,*f*). The addition of the replacement teeth follows the same sequence as the addition of the first-generation teeth, judging by the number of resorption surfaces beneath them. Thus, it seems that the replacement of each generation of teeth is a diachronous event that proceeds like a wave across the tooth cushion, starting at the labial margin; the functional dentition is at any one time composed of teeth of more than one generation, and the whole tooth field maintains functional teeth all the time.

The semi-basal resorption surfaces cutting off the first-generation teeth are fairly regular in shape (figure 9*c*). On top of the dentine ring the resorption surface takes the form of a circular platform that hardly extends out of the tooth into the surrounding bony tissue. Internally, it extends deeply into the base of the first-generation tooth (figure 12*d*). Hence the following generations of replacement teeth, along with their bone of attachment, can insert into the hollow, right at the same tooth site, and even the basal resorption surfaces of replacement teeth extend into the hollow too, like a funnel (figures 3–6, 8, 11 and 12*f*–*j*). But since the tooth cushion has grown wider and thicker, with a larger external surface, the replacement teeth do not overlap each other. The following generations of teeth even have to become larger and shift towards the lingual margin of the cushion in order to fill up the increasing space (figures 2*j*–11).

At least three or four resorption surfaces are stacked at each tooth site in the assumed lingual region of the thinner specimens (figures 4, 6*a*, 8–11) but more than 12 layers of resorption are exhibited in the assumed labial region of the thick one (figures 2*j*, 3 and 5*b*). No dentine remnant is found between these resorption surfaces, indicating that all the replacement teeth are shed completely by basal resorption. The resorption surface is centred on the predecessor tooth that was shed by the resorption event. The position, orientation and size of a resorption surface thus illuminates that of a shed tooth, and the stack of resorption surfaces reflects the track of the cyclical shedding. The position of the new tooth usually deviates from the resorption cup left by its predecessor, as it is larger and located more lingually (figures 8*b*,10*f* and 11*b*). Because both the semi-basal and basal resorption surfaces are funnel-shaped, this lateral displacement prevents them from ‘stacking’ neatly; each new tooth will be raised up, because it straddles the rim of the underlying resorption pit, and its bone of attachment will thicken the bony plate. Accordingly, the basal resorption of the new tooth will partially encroach the resorption surfaces the tooth rests upon, the resorption surfaces at the next more lingual tooth site, and even the buried dentine rings of the first-generation teeth (figures 8*b* and 11*b*; figure 10*b*–*e*, white arrow heads and arrow heads with black outline; figure 11*a*, *den), producing a new wider resorption surface. The encroachment by later resorption events is most obvious at the lingual side of the resorption surfaces or dentine rings located at the lingual margin, as the marginal replacement teeth incline lingually and this inclination is increasing in their successors. The bone of attachment sandwiched by the resorption surfaces is thus thicker at the labial side, and consists of the tissue between tooth sites (figure 11). However, the resorption surfaces never intersect between tooth rows, no matter how tightly they are arranged (figure 9*c*–*f*). It may imply that the developmental domain of tooth shedding of each tooth row is clearly demarcated.

## 5. Discussion

### 5.1. Identification of the tooth cushions

Before examining the significance of the cushions and their dentition in detail, we need to consider their precise identification. In some extant actinopterygians such as *Amia* [25] the entire oropharyngeal cavity internal to the marginal jawbones is covered with dermal bones bearing fields of teeth or denticles. Homologues of all these elements can be identified in sarcopterygian fishes such as the Devonian tetrapodomorph *Eusthenopteron* [25], indicating that the whole suite was present at the osteichthyan crown-group node. From external to internal, this suite comprises: (i) the inner dental arcade, containing the vomers, dermopalatines, ectopterygoids and coronoids, all moderately large bones with distinct individual morphologies, usually carrying spatially organized teeth; (ii) the entopterygoid and prearticular, large flat bones on the palatoquadrate and lower jaw covered with denticles; (iii) the parasphenoid and (if present) paraotic dental plates, moderately large flat or gently curved bones on the ventral face of the braincase, covered with denticles and (iv) the branchial dental plates, numerous small denticle-bearing elements on the gill arches. The tooth cushions of *Lophosteus* have been identified in the past as branchial dental plates [3], but we argue that this is based on superficial similarities and takes insufficient account of the total evidence.

Even though the *Lophosteus* material from Ohesaare comprises hundreds of specimens, all the non-marginal dental elements are tooth cushions of the type described here. There is no trace of large flat denticle-bearing bones, either entire or as fragments, or of any small elements that diverge from the stereotypic concavo-convex tooth cushion morphology. We conclude that the tooth cushions are the only dental elements in the oropharyngeal cavity of *Lophosteus*. The maxillate placoderms *Entelognathus* [14] and *Qilinyu* [15], which constitute the best outgroup with regard to this problem because they have marginal jawbones homologous with those of osteichthyans, have no dental elements in the oropharyngeal cavity at all. In *Lophosteus*, it is unlikely that tooth cushions occupied all the areas covered by categories (i)–(iv) in crown osteichthyans, because they do not show a degree of morphological variation commensurate with this hypothesis.

Taken together, the evidence suggests that the tooth cushions of *Lophosteus* covered only part of the oropharyngeal cavity. But which part? Clues are provided by their morphology (figures 1 and 2). The cushions carry relatively large teeth, organized in transverse files, rather than disorganized denticle fields. The growth centre of the cushion is always positioned asymmetrically, showing that it grew more in one direction than the other. The teeth are strongly inclined and point towards the direction of maximum growth. The edge of the cushion that shows least growth is often somewhat flattened. The asymmetric growth pattern suggests that one edge of the cushion was constrained, probably by proximity to another bone; if we assume that the teeth pointed inwards and/or backwards in the oropharyngeal cavity, to facilitate swallowing of prey, it follows that this constrained edge was external. This suggests that the cushions were positioned immediately internal to the marginal tooth-bearing bones [6] and formed an inner dental arcade. Further support for this hypothesis is provided by the concave internal surfaces, which demonstrates that each cushion rested on a projecting bump of endoskeleton. Palatoquadrates and Meckel's cartilages of early gnathostomes frequently show different kinds of ‘scalloping’ or surface undulation associated with attachment sites for the inner dental arcade or tooth whorls [26,27], but branchial arches do not.

Furthermore, branchial and pharyngeal dental plates display much greater morphological variation [28,29]. The teeth usually lack a strongly recumbent shape, and tend to be organized into multiple rows, instead of files as in the tooth cushions [25,30,31]. In most cases, a larger proportion of the bony base is devoid of teeth, while the dentigerous region is often composed of fused pedicels of bone of attachment, forming a lamina that may contact the branchial arch cartilage directly [32,33], and teeth can anchor within the cartilage through resorption by chondroclasts [34]. The teeth are attached by moderate [30] to tall [31] pedicels of bone of attachment, necessitated by the thick layer of soft tissue overlying the dental plate. In the tooth cushions, on the contrary, the whole external surface is covered with teeth, and the teeth are inserted into the bony base. The bony base of branchial dental plates, which only joins to a restrict area of the dentigerous lamina, may develop afterwards and independently of teeth [34]. Few of the root canals of the teeth are incorporated into the delicate bony base, while canals from the external surface of the bony base directly connect to the soft tissue, rather than through the tooth vascular system [32,33]. An intimate developmental relationship between the bony plate and the pharyngeal teeth seems not to be indispensible. The tooth replacement of the tooth cushion is more similar to that of the marginal jawbone [12] in terms of the gradual accretion of bone of attachment onto the external face of the basal plate during growth. These differences, while not dramatic, also argue against an identification of the tooth cushions of *Lophosteus* as branchial dental plates.

We conclude that the tooth cushions probably represent the inner dental arcade and are broadly homologous with the coronoids, vomers, dermopalatines and ectopterygoids of crown-group osteichthyans.

### 5.2. The dentition

The tooth cushions of *Lophosteus* exhibit tooth replacement by cyclic basal shedding, indicating that this osteichthyan autapomorphy has been acquired by both *Andreolepis* and *Lophosteus*, not only on the marginal jawbones but also on the inner dental elements. This unique osteichthyan trait emerges earlier than the presence of enamel on the teeth, as tooth enamel is absent not only in *Andreolepis* and *Lophosteus*, but also in the more derived stem osteichthyan or basal crown osteichthyan *Psarolepis* [3,12,35,36].

The teeth on the tooth cushion are not randomly distributed in an irregular tooth field, but neatly arranged in tooth files. Thus the tooth cushion could be described as a multi-file tooth whorl or tooth plate. On the tooth whorls lining the jaw or in the symphysis of acanthodians and early chondrichthyans, successive teeth of increasing size are added lingually [37,38]. The post-functional teeth are rotated labially out of the mouth [39], merging into the facial ornamentation [40] or incurving spirally. The tooth plates of crown-group holocephalans are formed by fusion between crowns as well as bases of a few tooth families [41], with the new teeth stacked up against the post-functional teeth. In spite of retention of the compound teeth, new tooth material is added on one side as the teeth are abraded on the other, so that the material of the tooth plate travels diagonally from lingual to labial for a substantial distance during the lifespan of the individual, even though the tooth plate as a physical object remains in one place [42]. The mechanisms of constant rotation and lingual addition of new teeth are also seen in the parasymphysial tooth whorls of sarcopterygians, such as *Onychodus* and porolepiforms. However, as these dental elements are internal to the dentary bone, which forms the jaw margin, their post-functional teeth cannot be rotated out to the face. Neither end of the whorl is static. New teeth that are fully grown will be inserted by the extension of the posterior (i.e. lingual) end of the whorl, and post-functional teeth will be lost by basal resorption immediately before the resorption of the anterior end of the whorl itself [43,44]. Shedding by basal resorption is lost in the tooth plates of lungfish. Sets of teeth are added on non-alternate radiating rows from the initial tooth, but instead of site-specific cyclic resorption, worn teeth may be removed by extensive resorption, before being replaced by denticles or sheet dentine [45]. All these tooth systems have been interpreted as generated by a dental lamina.

However, a single lingually positioned dental lamina of the kind seen in sharks cannot explain the tooth addition and replacement mechanism of the tooth cushion. On the tooth cushion of *Lophosteus*, all the tooth sites are active throughout life. There is no post-functional position (away from the biting region) that the worn teeth can be moved to. Although continued growth is observed at the active margin of the tooth cushion even when the number of tooth sites does not increase any more, neither significant bone resorption or abrasion at the primary zone, nor movement of the functional sites associated with the remodelling of the basal plate is observed. The basal plate of the cushion is relatively static. Resorption only relates to particular teeth and the teeth can only be renewed via *in situ* replacement. Furthermore, the older tooth sites undergo more cycles of replacement than the younger ones (figures 3–6), thus teeth initiated at the same time may be of different generations at different tooth sites (figure 10*e*,*f*). If the tooth buds of one generation are all generated by a single, lingually positioned dental lamina, a marginal functional tooth will block the addition of the next generation of teeth to the preceding tooth sites. This suggests that each tooth site has its own clock of replacement and the cyclic shedding is autonomous. In addition, there is no space for a proper dental lamina to situate deeply at the side of each tooth site, considering that the replacement teeth are added superficially, similar to those on the jawbone of *Andreolepis* [12]. The replacement tooth bud is more likely to form directly from the dental epithelium of the predecessor tooth, or from a small successional dental lamina associated with that individual tooth, as in the majority of actinopterygians [46–48]. All these data and inferences point to the same conclusion that the dentition on the tooth cushion of *Lophosteus* does not depend on a permanent dental lamina, and give further support to the emerging consensus that the permanent dental laminae of chondrichthyans and tetrapods are convergent [12,49–51].

What makes the tooth cushion of *Lophosteus* distinctive relative to all other dental elements known so far is the combination of semi-basal and basal resorption. While the post-functional teeth of other tooth whorls or tooth plates are either shed or not, the first-generation teeth of *Lophosteus* tooth cushion are shed semi-basally. The resorption actually extends deep down to the vascularized base of the tooth, in order to allow the replacement teeth to insert into the ring of dentine remnant (figures 11 and 12). The resorption resistance of the tooth base, which is rich in osteocytes, may be due to the inactivity of osteoclasts, and the inability of the odontoclasts to resorb bone of attachment. Another potential reason may be the embedding of the tooth base and bone of attachment in the newly grown bony tissue, as the bony base has been thickened during the addition of the first-generation teeth. As the first-generation teeth would be shed before the addition of the next tooth in the same radial tooth file, they could only perform food processing for a relatively short time, after which the job would be carried on by a cyclic shedding dentition with proper basal resorption at the same sites. Therefore, the principal function of the first-generation teeth was probably to establish the fixed tooth sites for the successor teeth, patterning the tooth organization.

The semi-basal shedding nature of the first-generation teeth of the *Lophosteus* tooth cushion is reminiscent of the non-shedding first-generation odontodes on the marginal jawbone of *Andreolepis* [12]. However, these odontodes undergo irregular apical resorption occasionally, and are overgrown by gap-filling odontodes, in a manner reminiscent of its scale odontodes [11]. The shedding dentition is established at the lingual margin of the first-generation odontodes [12], whereas on the tooth cushion of *Lophosteus* the first-generation teeth directly set up the shedding dentition *in situ*. Although the semi-basal resorption cannot remove the entire teeth, it is site-specific and initiated from inside. This is presumably because the odontoclasts, probably the only clast cells involved in the semi-basal resorption, differentiated within the pulp cavity of a tooth and only affected the dentine of this tooth. Semi-basal resorption might be an initial form of osteichthyan-type tooth resorption, and it may have evolved into a real basal resorption by recruiting osteoclasts to resorb the bone of attachment of the tooth, as displayed by the successive generations of replacement teeth on the tooth cushion of *Lophosteus*.

The pattern of addition of first-generation odontodes on the marginal jawbone of *Andreolepis* and first-generation teeth on the tooth cushion of *Lophosteus*, both resemble the addition of teeth in non-shedding dentitions such as the jawbones of arthrodires and ischnacanthid acanthodians [52], which may demonstrate that tooth addition along with the growth of basal bone is the primitive state for jawed vertebrates. Tooth shedding and replacement emerged independently in osteichthyans and chondrichthyans. The combination of non-shedding or semi-shedding with shedding dental systems could be a transitional feature distinctive for stem osteichthyans, even though it might not be shared by all the stem members. The tooth cushion of *Lophosteus* provides a clue about the evolutionary relationship between non-shedding tooth addition and osteichthyan-type tooth replacement. Especially, it casts light on the origin and the development of the tooth fields internal to the marginal jawbones of osteichthyan fish.

## Data accessibility

All original data will be made available through the ESRF palaeontology database (http://paleo.esrf.fr/picture.php?/2300/category/1765/created-monthly-list-2014-7).
